# Percutaneous Intramedullary Application of Stem Cells for Fifth Metatarsal Fractures Treated With a Cannulated Screw

**DOI:** 10.7759/cureus.55185

**Published:** 2024-02-28

**Authors:** Nikolaos E Koukoulias, Evangelia Germanou, Dimitris Koukoulias, Theodoros M Kannas, Thefilos Dimitriadis

**Affiliations:** 1 Department of Sports Trauma and Orthopaedics, St. Luke's Hospital, Thessaloniki, GRC; 2 Department of Physical Education and Sport Science, Aristotle University of Thessaloniki, Thessaloniki, GRC; 3 Department of Physiotherapy, International Hellenic University, Thessaloniki, GRC; 4 Laboratory of Neuromechanics, Department of Physical Education and Sport Science, Aristotle University of Thessaloniki, Thessaloniki, GRC; 5 Department of Sports Trauma and Orthopaedics, St. Luke’s Hospital, Thessaloniki, GRC

**Keywords:** non-union, jones fracture, fracture healing, fifth metatarsal, stem cells

## Abstract

Non-union and refracture of fifth metatarsal fractures are common and devastating complications in the athletic population. Stem cell application at the fracture site, for biologic enhancement, is utilized to address this challenge. We present a simple technique to approach both the endosteum and the periosteum percutaneously, under a local anesthetic, in cases of cannulated screw intramedullary fixation.

## Introduction

Fracture of the fifth metatarsal base is the most common foot fracture seen in professional and recreational athletes [1,2]. Surgical treatment yields superior healing rates compared to conservative treatment for this group of patients [1,2]. For the Zone II (Jones fracture) and Zone III (stress fracture) types, intramedullary screw fixation is considered the standard method of treatment [1].

Nevertheless, up to 20% of non-union rate can be encountered [1]. Moreover, a 5.6% refracture rate has been reported, despite internal fixation [2] while one study reported a 30% refracture rate in elite athletes [3].

This devastating complication has been attributed to early return to play [4], non-optimal biomechanics [1], and limited inherent blood supply in this area [5]. As a result, biologic augmentation with stem cell utilization has been proposed, either initially, during fixation as an adjacent, or subsequently after the complication has occurred [1].

So far, stem cell application has been either performed open as part of a revision surgery or percutaneously close to the periosteum [1]. The open approach is more traumatic and requires hospitalization while the traditional percutaneous approach has no access to the intramedullary canal and endosteum. Both periosteum and endosteum contribute to callus formation and as a result, regeneration strategies should target both anatomic areas [6,7].

We present a simple and consistent way to approach the intramedullary canal percutaneously, under a local anesthetic.

## Case presentation

We present a case of a 22-year-old, male, professional basketball player who suffered a proximal fifth metatarsal fracture. The patient felt an acute pain in his right foot after landing from a jump, during practice. The patient was a non-smoker and had a clear medical history.

**Figure 1:**
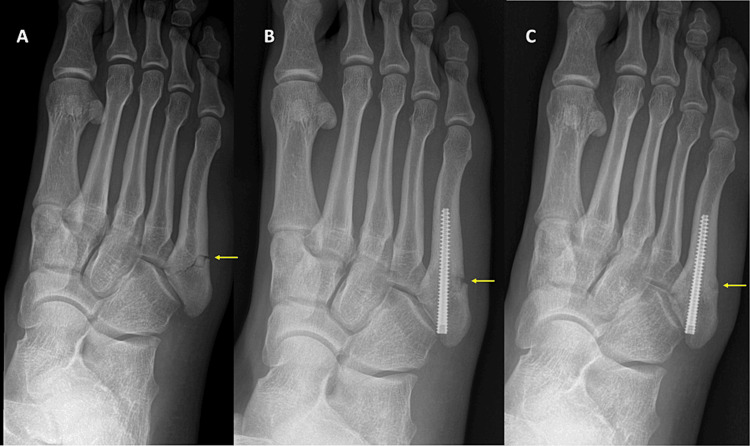
Fifth metatarsal non-union. The yellow arrow points to the fifth metatarsal fracture site. A. Jones fracture in a pro athlete. B. Non-union of Jones fracture 12 weeks after intramedullary cannulated screw fixation. C. Healing of the fracture four weeks after percutaneous stem cell application.

Clinical examination revealed proximal fifth metatarsal tenderness and edema. The athlete was referred for imaging studies. Radiographic evaluation of the foot revealed a Jones fracture (Figure 1A). Due to his athletic profile, surgical treatment was planned, and intramedullary screw fixation was performed two days after the injury. An Acutrak® 4/5, 45 mm in length screw (Acumed, LLC, Hillsboro, USA) was inserted percutaneously under fluoroscopy. Post-operatively, the foot was immobilized in a walking boot and a structured rehabilitation regime was initiated. Partial weight bearing with crutches was allowed according to pain tolerance and range of motion exercises initiated one month after surgery. The patient was completely asymptomatic, with full weight bearing and full range of motion at six weeks post-operatively and the boot was discontinued. Nevertheless, radiographic evaluation demonstrated uncompleted fracture healing (Figure 1B) and the patient was not permitted to return to unrestricted athletic activities. Core and upper limb exercises were allowed along with open chain lower limb exercises in order to protect the healing process. Follow-up radiographic evaluation showed non-union of the fracture at twelve weeks post-operatively and biologic enhancement of the healing process with stem cells was decided. The option of revision, open internal fixation with bone grafting was rejected at this stage, due to the morbidity of the procedure. The fracture was found healed at four weeks post-stem cell application (Figure 1c).

For the treatment of fifth metatarsal fracture non-union with stem cells, the patient is positioned in the supine position, and the involved foot is prepped and draped. The skin and soft tissue proximal to the base of the fifth metatarsal, along with the area around the fracture site, is anesthetized with lidocaine 2%. 

The stem cells are prepared according to the surgeon’s preferred technique. In our case, we used the MarrowStim Concentration System® (Biomet Biologics, Warsaw, U.S.). Fourteen mls with 30.3x106 cells/ml were injected in total. A spinal needle (14Gx2") is utilized to deliver the stem cells to the fracture site. 

**Figure 2:**
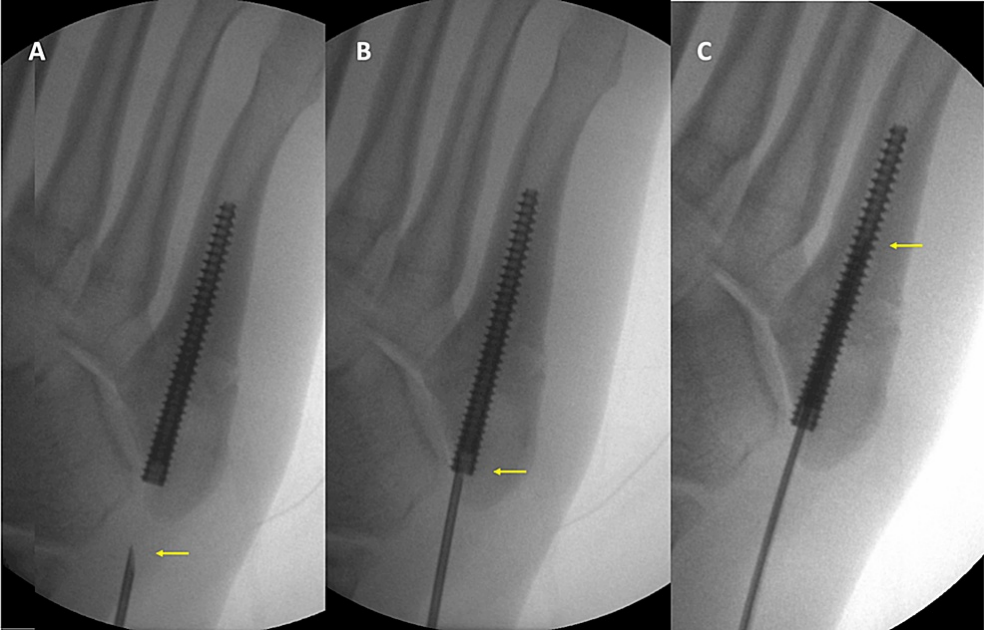
Needle advancement. The yellow arrow points the tip of the needle. A. The needle is guided toward the base of the fifth metatarsal. B The base of the cannulated screw is used as the entry point of the medullary canal. C. The needle is advanced into the medullary canal.

**Figure 3:**
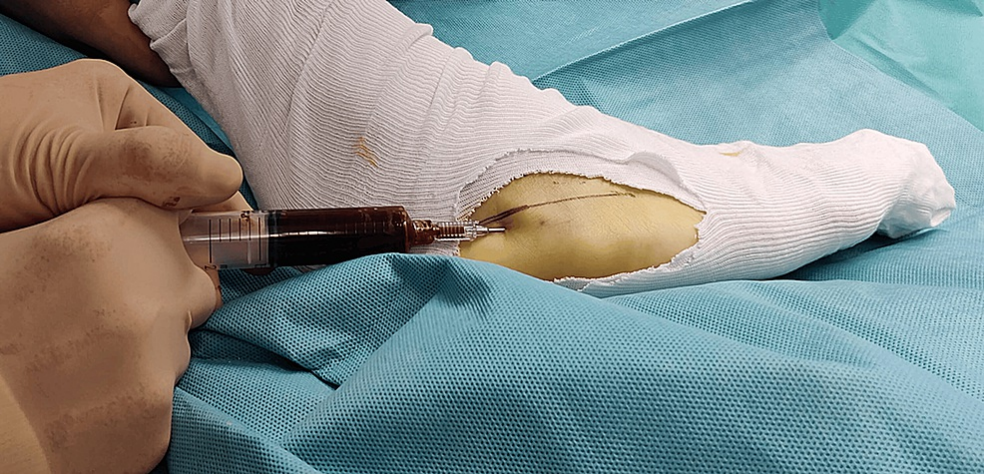
Percutaneous, intramedullary application of stem cells. After the optimal position of the tip of the needle has been confirmed, the stem cells are released.

The stem cells were applied percutaneously under a local anesthetic with the aid of an image intensifier. The needle is introduced through the skin incision that has been previously used for the insertion of the cannulated screw. The tip of the needle is then guided to the base of the cannulated screw (entry point of the medullary canal) and the trajectory of the needle is aligned to the axis of the screw. The needle is then advanced into the cannulated screw (Figure 2) and the stem cells are released into the medullary canal (Figure 3).

As the stem cells fill the medullary canal, the intramedullary pressure rises and the surgeon feels an increased resistance, meaning that the intramedullary application of stem cells has been completed. In total, 7 mls (half of our solution) were injected into the intramedullary canal without having any leakage through the soft tissues in our case. 

**Figure 4:**
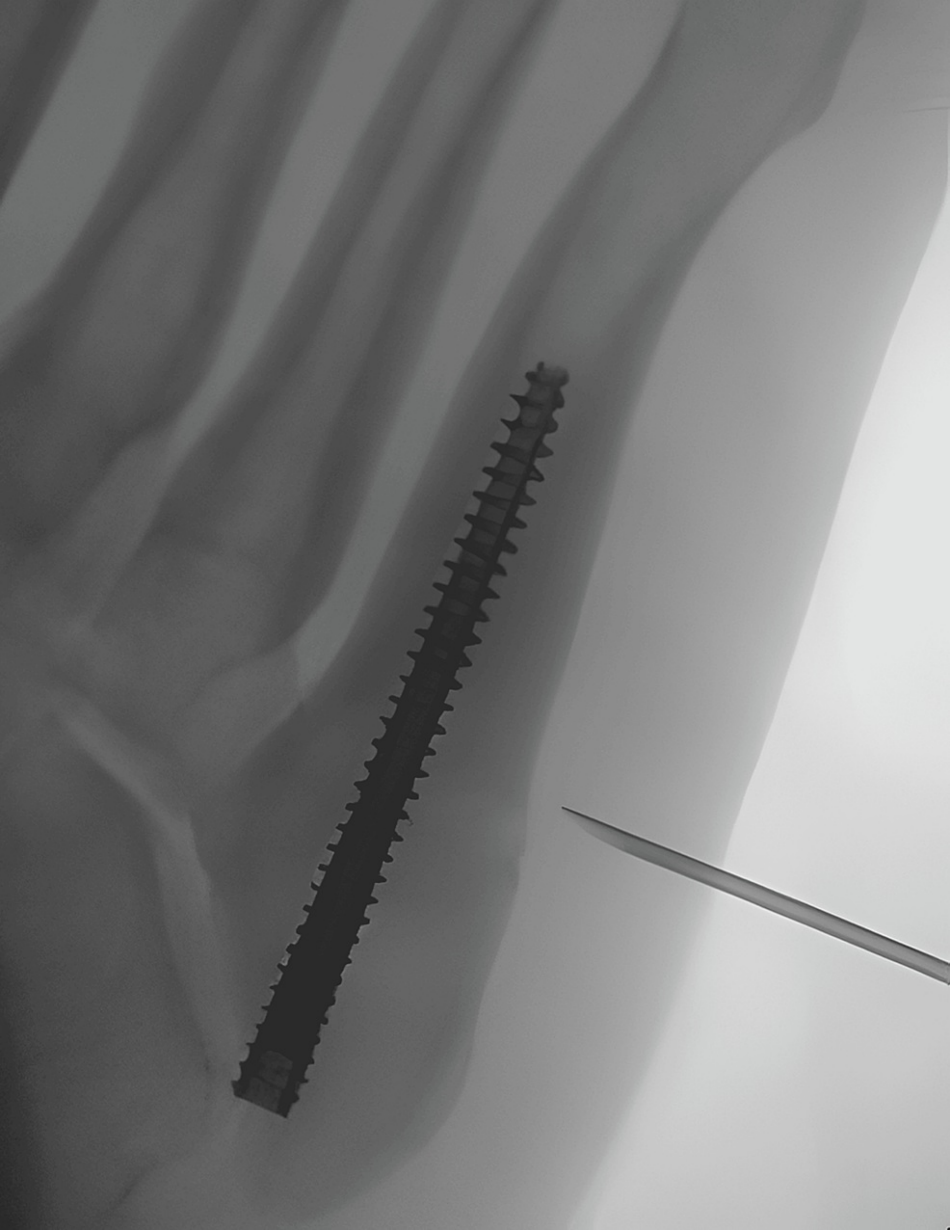
Percutaneous, extramedullary application of stem cells. The tip of the needle is guided close to the fracture area under fluoroscopic control.

The needle is then completely withdrawn and a new needle (14G, 2") is applied to the syringe with the stem cells. The needle is then positioned close to the periosteum at the fracture site, under fluoroscopic control (Figure 4).

Once the optimal position of the tip of the needle is confirmed, the remaining stem cells (7mls in this case) are released extramedullary, next to the fracture site. The surgeon can reposition the tip of the needle several times as the type of fracture requires.

No foot range of motion and weight-bearing restrictions were applied and the athlete continued his rehabilitation regime. The fracture was found healed at four weeks post-stem cell application (Figure 1c) and the patient was allowed to return to sports. Two years after his return, the athlete remains asymptomatic and fully active.

## Discussion

The low success healing rate and unpredictable outcome of proximal fifth metatarsal fractures have raised interest in biologic augmentation. Application of stem cells and PRP [1,6,7], grafting of the fracture [8], addition of pulsed electromagnetic field [9], and vitamin D supplementation [10] have been used to stimulate bone healing. There is no direct comparison of the different biologic augmentation techniques.

Stem cells are probably the most popular and effective way to promote healing both during initial fixation or in case of non-union or refracture [1,6,7]. The efficacy of stem cell application for the treatment of non-union has been proven in animal studies [11]. Hunt and Anderson [12] used stem cells along with demineralized bone matrix (DBM) grafting to treat refractures or non-unions in athletes and they reported zero failures in eight athletes. O'Malley et al. studied 10 NBA players with fifth metatarsal fractures [3]. For this demanding study population, they used percutaneous screw intramedullary fixation along with prophylactic stem cell application. In three athletes they additionally used open grafting of the fracture site. They reported radiographic healing at an average of 7.5 weeks and return to play at 9.8 weeks. However, they encountered three refractures in their series [3].

As a result, it is clear that optimal treatment of fifth metatarsal fractures, non-unions, and refractures in athletes has not been established yet [1,2]. Both prophylactic [3] and therapeutic [12] utilization of stem cells has been proposed. The first advantage of our technique is the simplicity, which allows straightforward application during surgery, or later in case of non-union under local anesthetic. 

From the biological perspective though, the main advantage of our technique is the intra- and extramedullary application of stem cells at the fracture area. Both endosteum and periosteum contribute to the healing process and biologic enhancement of both sites is essential [6,7]. Conventional percutaneous application cannot reach the intramedullary canal and may be insufficient in providing a favorable healing environment [8]. We have to acknowledge though that evidence of stem cells' presence between the screw and the bone was not presented in this technical note. However, the injection of 7 mls in the medullary canal, along with the feeling of high pressure during injection, ensures the filling of the medullary canal with the stem cells. Moreover, the helicoid form of the threads of the screw provides space to accommodate the stem cells between the screw and the endosteum and access for them to reach the fracture site.

Another advantage of our technique is the avoidance of hospitalization and open approach. As a result, the cost of this procedure is minimized and the patient can immediately resume his level of activities without adding any extra pain or morbidity.

It is also a versatile and totally safe technique. The surgeon can elect to use it along with any other biologic augmentation technique that is required with no restrictions. The fact that no extra incision or approach is required ensures that there is no extra risk of neurovascular complications.

This technique has also been proven effective in our hands with complete fracture healing in four weeks (Figure 1) after application. Due to our positive experience with this technique, we use it as a first-line treatment in case of non-union or refracture and we also use it prophylactically in high-demand patients, like professional athletes, to avoid delayed-union or non-union.

The main disadvantage of the technique is the fact that is only applicable with intramedullary cannulated screw fixation. Our technique cannot be employed when solid screws or plates have been used, because the intramedullary canal is not accessible.

## Conclusions

In conclusion, percutaneous application of stem cells, through the cannulated screw, into the medullary canal is a simple, safe, and effective technique for fifth metatarsal fractures. The surgeon can apply this technique during initial fixation with a cannulated screw to minimize the healing time or in case of non-union or refracture.
